# Qi Fu Yin–a Ming Dynasty Prescription for the Treatment of Dementia

**DOI:** 10.1007/s12035-018-0908-0

**Published:** 2018-02-07

**Authors:** Wei-Yi Ong, Ya-Jun Wu, Tahira Farooqui, Akhlaq A. Farooqui

**Affiliations:** 1 Department of Anatomy, National University of Singapore, Singapore, 119260 Singapore; 2 Neurobiology and Ageing Research Programme, National University of Singapore, Singapore, 119260 Singapore; 3 Department of Molecular and Cellular Biochemistry, The Ohio State University, Columbus, OH 43220 USA

**Keywords:** TCM, Dementia, Ginseng, Rehmannia

## Abstract

The Traditional Chinese Medicine (TCM) theory that “kidneys give rise to marrow, and the brain is the sea of marrow” has been a guide for the clinical application of kidney, qi and blood tonics for prevention and treatment of dementia and improvement in memory. As low resistance end-organs, both the brain and the kidneys are subjected to blood flow of high volumes throughout the cardiac cycle. Alzheimer’s disease and vascular dementia are two common causes of dementia, and it is increasingly recognized that many older adults with dementia have both AD and vascular pathologies. The underlying molecular mechanisms are incompletely understood, but may involve atherosclerosis, vascular dysfunction, hypertension, type 2 diabetes, history of cardiac disease and possibly, kidney dysfuntion, leading to reduced erythropoietin production, anemia, brain energy deficit and slow excitotoxicity. During the Ming Dynasty, Zhang Jing-Yue used Qi Fu Yin (seven blessings decoction), comprising *Panax ginseng*, *Rehmannia glutinosa*, *Angelica polymorpha*, *Atractylodes macrocephala*, *Glycyrrhiza uralensis*, *Ziziphus jujube*, *and Polygala tenuifolia* to boost qi and blood circulation, strengthen the heart, and calm the spirit—skillfully linking heart, spleen, kidney, qi, blood and brain as a whole to treat age-related dementia. The purpose of this review is to outline TCM concepts for the treatment of dementia and illustrated with a historical prescription for the treatment of the condition, with the hope that this description may lead to advances in its management.

## Introduction

**Fig. 1:**
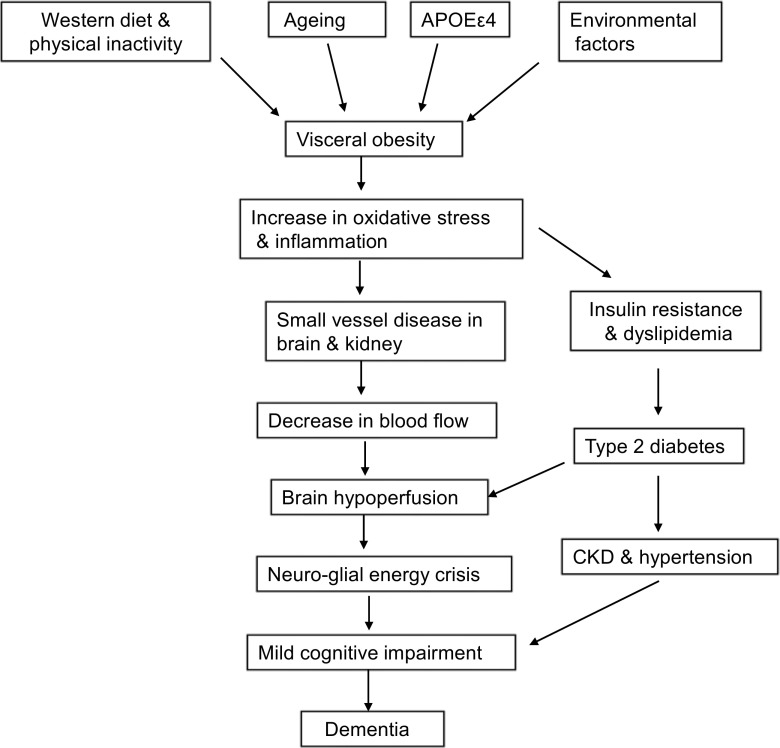
Factors contributing to the pathogenesis of chronic kidney disease (CKD) and dementia

Dementia is a progressive syndrome characterized by memory deficits, cognitive impairment, and deterioration in emotional control and social behavior [1, 2]. The loss in cognitive function is more than what is typically experienced in normal aging, and results from damage caused by the disease process, such as Alzheimer disease (AD), Parkinson disease (PD), and traumatic brain injury (TBI) [3]. Several types of dementia have been reported, including vascular dementia, progressive dementia, Lewy body dementia, Alzheimer’s dementia, and dementia as a result of diseases such as stroke, AIDS, or multiple sclerosis [4]. Age, prolonged consumption of “Western” diet, and physical and mental inactivity, as well as environmental factors are major risk factors that predispose one to dementia (Fig. 1). Other factors include cardiovascular and cerebrovascular complications, excessive alcohol intake, social isolation, prior head injury, and the possession of a single or two copies of the APOEϵ4 genetic variant [1, 2, 3]. The symptoms of dementia may be linked with alterations in neuroplasticity in corticolimbic brain regions. In particular, divergent responses have been reported; neuronal atrophy and synapse loss in the prefrontal cortex and hippocampus, and neuronal hypertrophy and increased synaptic density in the amygdala and nucleus accumbens are found [5]. Mild cognitive impairment (MCI) is a pathological condition of modest cognitive decline that does not interfere with one’s ability to perform activities of daily life, and is considered to be a symptomatic pre-dementia state [6]. Not everyone with MCI will progress to dementia, but individuals with MCI develop dementia at higher rates than those with normal cognition [6, 7].

**Table 1:**
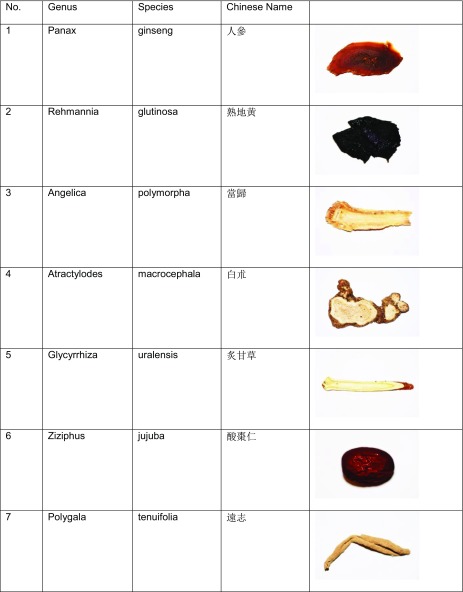
Herbs used in Qi Fu Yin

The prevalence of dementia increases exponentially with age, from 3.0% among those aged 65 to 74 years to 18.7% among 75 to 84 years old and 47.2% in individuals over the age of 85 years [8]. The worldwide prevalence of dementia is estimated to double every 20 years and reach over 81 million by 2040, due to an aging population [9]. An understanding of molecular mechanisms that contribute to the pathogenesis of dementia is essential for successful interventions. Dementia, like neurodegenerative diseases, cardiovascular disease, and cancer, requires early detection to potentially arrest or prevent further disease progression. The purpose of this review is to outline Traditional Chinese Medicine (TCM) concepts for the treatment of dementia and illustrated with a historical prescription for the treatment of the condition, with the hope that this description may lead to advances in its management.

## TCM Ideas of Brain and Treatment of Dementia

According to TCM, the brain and bone marrow are the outgrowths of the kidneys. In Lingshu Meridians, it is stated that “at conception, essence is formed.” After essence is formed, the brain and bone marrow are formed. The kidneys contain the essence, the essence sustains the marrow, and the marrow nourishes the brain. According to “Lingshu Discussion on Seas,” humans have a marrow sea, a blood sea, a qi sea and a water/grain sea (stomach). This is the meaning of the four seas. Among the four seas in the human body, the marrow sea refers to the brain. According to the “Category Text” Volume 9: where there is bone, there is marrow, and the brain has the most. Thus, all marrow is related to the brain, and the marrow sea refers to the brain. <《灵枢·经脉》曰:“人始生,先成精,精成而脑髓生。” 肾藏精,精充髓,髓荣脑。《灵枢·海论》歧伯曰:“人有*髓海,有血海,有气海,有水谷之海,凡此四者,以应四海也。”人体四海之一,髓海即指脑。《类经》卷九注:“凡骨之有髓,惟脑为最巨,故诸髓皆属于脑,而脑为髓之海。> [10, 11]. This, TCM theory of “kidneys give rise to marrow, and the brain is the sea of marrow” has been a guiding principle behind traditional Chinese medicine’s clinical utilization of qi, blood and kidney tonics for the prevention/ treatment of dementia and improvement of memory for thousands of years. < 肾生髓,脑为髓海”的理论指导中医临床应用补充气血兼补肾中药来防治痴呆和提高记忆能力已经有数千年的历史。> [10] . Thus, in TCM, nourishing agents, such as Rehmannia; kidney essence astringents, such as rose fruit; and blood tonics such as angelica are used to benefit the brain. Memory and cognition are disordered if channels that connect the heart and brain are blocked by phlegm; hence, herbs such as zizyphus and polygala are also used to improve heart function, together with Qi tonic herbs such as ginseng [12].

## TCM Ideas of Qi and Phlegm that Blocks Qi

TCM views the body as a single entity and an individual’s being depends on interactions between the different body parts. Qi, blood, and body fluids are essential for life. Qi is interpreted as “life energy” or “life force.” The Chinese character for “qi” (氣) means air or gas and may have some of the characteristics of air or oxygen gas. Original qi originates from the kidneys, the site where “congenital essence,” an essential and vital substance inherited from one’s parents upon conception is stored. Pectoral qi is formed from the combination of inhaled fresh air from the lungs and food essence from the stomach and spleen. It permeates the blood vessels and moves outward during expiration and inward during inspiration. Nutritive qi supplies nourishment to the body. It comes from food and digestive activities and circulates via blood vessels. Protective qi is similar to the immune system and helps in the prevention of illnesses.

Phlegm is a pathological substance generated by disturbance of body fluid that blocks qi. Substantial phlegm is visible such as sputum, whereas insubstantial phlegm is invisible. Lipids and lipoprotein metabolic disorders (atherosclerosis, diabetes and metabolic syndrome) are believed to contribute to phlegm in the blood, whereas platelet activation, thrombosis, endothelial injury, and atheromatous plaques contribute to blood stasis [13]. Small-molecule substances such as inflammatory factors (cytokines, chemokines, and platelet-activating factor) and arachidonic acid-derived enzymatic (prostaglandins, leukotrienes, and thromboxanes) and non-enzymatic (4-hydroxy-2-nonenal, isoprostanes, isofurans, isoketals, and acrolein) lipid mediators and free radicals (oxygen radicals, superoxides, and hydroxyl radicals) likely play a role in phlegm and blood stasis.

## Physiological and Pathological Connections Between the Kidneys and Brain

The TCM theory of “kidneys give rise to marrow, and the brain is the sea of marrow” implies a close relationship between the kidneys, the marrow, and the brain. Chronic renal failure is asymptomatic at first, until kidney function has decreased to less than 25% of normal. Patients then present with nocturia and anorexia, and raised serum levels of nitrogenous compounds such as urea and creatinine. Advanced renal failure causes significant impairment of all renal function and affects virtually all body systems, and causes change in urea, electrolytes, and other blood constituents. End-stage renal disease is the term used when more than 90% of renal function is lost and may be complicated by anemia, bleeding, bone disease, hypertension, congestive heart failure, digestive tract problems, and dementia. It is proposed that part of the renal-cerebral connection may be due to small vessel disease in both the kidneys and brain. There are many hemodynamic similarities between the vascular beds of these organs [14]—both kidneys and brain are low resistance end-organs and subjected to blood flow of high volume throughout the cardiac cycle unlike those of other body organs [15]. Abnormalities in capillaries are found in the kidneys and brains of people who die of dementia, and similar capillary findings are observed in the kidneys of people with albuminuria, leading investigators to predict that albuminuria and small vessel disease of the brain go hand in hand [14, 16]. Patients with albuminuria have about 50% greater chance of developing dementia than people without albuminuria [17]. Persistent albuminuria indicates that the kidneys are damaged and starting to spill albumin into the urine. This supports the view that early detection and treatment of albuminuria and kidney disease may be important for protecting brain function. Drugs commonly used for the treatment of high blood pressure, e.g. ACE inhibitors and angiotensin-receptor blockers, may have a protective effect on the kidneys [16, 18]. Chronic kidney disease (CKD) patients are also often exposed to traditional risk factors, such as older age, hypertension, diabetes, and hyperlipidemia, as well as non-traditional risk factors, such as hyperhomocysteinemia, oxidative stress, and inflammation, that are associated with cognitive impairment in the general population [19, 20]. Oxidative stress and changes in redox status are closely associated with the pathogenesis of CKD. Both these processes increase the risk of stroke, induction of cognitive impairment, and onset of dementia in the aging brain [21, 22] (Fig. 1).

**Fig. 2:**
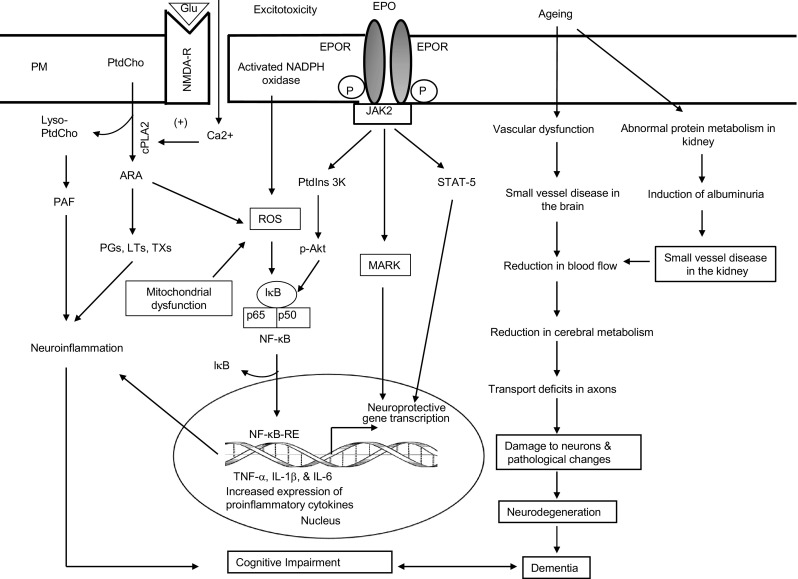
Hypothetical diagram showing interactions between excitotoxicity and erythropoietin receptor signaling along with the contribution of aging on dementia. Plasma membrane (PM); glutamate (Glu); N-methyl-D-aspartate receptor (NMDA-R); phosphatidylcholine (PtdCho); lyso-phosphatidylcholine (lyso-PtdCho); cytosolic phospholipase A_2_ (cPLA_2_); arachidonic acid (ARA); cyclooxygenase-2 (COX-2); 5-lipoxygenase (5-LOX); prostaglandins (PGs); leukotrienes (LTs); thromboxanes (TXs); platelet-activating factor (PAF); reactive oxygen species (ROS); nuclear factor kappa B (NF-κB); inhibitory form of nuclear factor kappa B (IκB/NF-κB); nuclear factor κB-response element (NF-κB-RE); inhibitory subunit of NF-κB (IκB); tumor necrosis factor-α (TNF-α); interleukin-1β (IL-1β); interleukin-6 (IL-6); Janus kinase 2 (JAK2); erythropoietin (Epo); erythropoietin receptor (EpoR); phosphatidylinositol 3Kinase (PtdIns 3K); serine/threonine kinase (Akt); Signal transducer and activator of transcription 5 (STAT5); and mitogen-activated protein kinases (MARK)

Anemia is a common finding in patients with CKD. Analyses of baseline data from adults with chronic kidney disease indicate that the prevalence of cognitive impairment is higher among those with lower eGFR, and this association is independent of traditional vascular risk factors, but rather, related to anemia [23]. The kidneys produce a glycoprotein hormone, erythropoietin (Epo), in response to hypoxia. It stimulates the production of red cells in the bone marrow and has been used for the treatment of anemia in humans [24]. Epo acts through a specific erythropoietin receptor (EpoR) on the surface of red cell precursors in the bone marrow and facilitates their transformation to mature red blood cells. Kidney dysfuntion may therefore lead to anemia, brain energy crisis, and slow excitotoxicity. Recent study shows anemia is associated with risk of dementia in older adults [25]. This could be a basis for the link between the kidneys, marrow, and brain in TCM theory. Epo and its receptor signaling via JAK2 activate multiple downstream signaling pathways including STAT5, PtdIns 3K/Akt, NF-κB, and MAPK (Fig. 2) [26]. These pathways are not only associated with red blood cell proliferation but also vasodilation [27] and insulin-sensitization [28].

Epo and EpoR are also expressed in neurons of the CNS, including the neocortex, hippocampus, and hypothalamus, as well as dorsal root ganglia, and Schwann cells [29]. Epo improves sensorimotor and spatial learning functions, and peripherally administered Epo crosses the blood-brain barrier and stimulates neurogenesis and neural differentiation [30, 31]. Activation of NF-κB signaling contributes to EPO-mediated neuroprotection. During the “classical” activation of NF-κB, phosphorylation of inhibitor of NF-κB (IκB) by IκB kinase (IKK) leads to degradation of IκB by ubiquitination, NF-κB translocation to the nucleus and induction of gene expression. In contrast, after Epo treatment of neural cells, JAK2 phosphorylate IκB resulting in ubiquitination-independent dissociation of IκB from the NF-κB complex, translocation of NF-κB to the nucleus, and induction of neuroprotective genes [32]. NF-κB promotes neuroprotection via inhibitor of apoptotic protein (IAP), which reduces tumor necrosis factor-α (TNF-α) [33] and apoptotic JNK signaling [34], and activates Bcl-x_L_ [35]. In addition, activation of JAK kinases results in tyrosine phosphorylation and dimerization of STAT, which translocates to the nucleus and binds to specific DNA sequences in the promoters of responsive genes to promote gene transcription supporting neurogenesis and angiogenesis [36]. Collective evidence suggests that in the brain, Epo mediates neurotrophic, anti-apoptotic, antioxidant, and anti-inflammatory effects [37].

CKD patients also show raised serum levels of nitrogenous wastes. Decreased cognitive function has been associated with the retention of uremic toxins, with performance improving with more intensive dialysis or kidney transplantation [38]. The enzyme glutamine synthetase (l-glutamate:ammonia ligase (ADP-forming; E.C.6.3.1.2) is located in astrocytes and catalyzes the reaction of glutamate with ammonia to form glutamine [39, 40]. It is part of the glutamate-glutamine shuttle that is essential for normal glutamatergic neurotransmission. High levels of ammonia interferes with this shuttle, and increased serum ammonia is associated with symptoms such as impaired memory, shortened attention span, sleep-wake inversions, brain edema, intracranial hypertension, seizures, ataxia, and coma [41, 42]. This is a major factor in hepatic encephalopathy as a result of failure to metabolize ammonia to urea in liver disease, but less is known about whether it could have a role in CKD. Nevertheless, it is conceivable that failure of urea excretion as a result of kidney dysfunction could lead to buildup of ammonia and affect the brain via a similar mechanism.

## Physiological and Pathological Connections Between the Circulatory System and Brain

The TCM theory that memory and cognition are disordered if channels that connect the heart and brain are blocked by phlegm implies a close relationship between the cardiovascular system and the brain, a connection which is also highly emphasized in Western medicine. Heart disease is any condition that impairs cardiac function regardless of the specific modality that is affected [43]. The functionality of other organs, including that of the brain is at risk in patients with heart disease [44]. There is an expanding body of literature indicating that heart disease and atrial fibrillation are risk factors for dementia [43, 45]. Additionally, several studies suggest that cardiovascular risk factors are independently associated with the development of dementia. These include hypertension, hypercholesterolemia, type 2 diabetes, obesity smoking, and elevated plasma levels of homocysteine [46, 47]. Reduced brain perfusion and reduced oxygen and/or glucose supply or utilization by the brain could cause an energy crisis in neurons and affect the function of ion pumps such as Na^+^/K^+^ ATPases that maintain resting potential in neurons. This results in depolarization, excessive calcium entry into neurons, activation of calcium dependent enzymes such as proteases, lipases, and endonucleases, and slow excitotoxicity [48]. Reduction in cerebral blood flow due to heart disease may also magnify any cognitive problems caused by the accumulation of endogenous toxic products such as ammonia, lactic acid, nitric oxide, proinflammatory eicosanoids, cytokines and chemokines. Accumulation of these molecules may contribute to the pathogenesis of heart disease, ischemic stroke, dementia, and AD. Reduced brain perfusion could also affect the clearance of amyloid beta (Aβ) and hyperphosphorylation of Tau protein, inducing the formation of amyloid beta plaques, neurofibrillary tangles, atherosclerosis, and inflammatory cascade (Fig. 1). These processes not only increase the risk of immune exhaustion [49], but are also magnified by type 2 diabetes, obesity, and hypertension [50]. They may not only induce synaptic dysfunctions and impairment in cerebral flow leading to dementia and AD [51, 52], but also disrupt the BBB and affect the process of neurogenesis [53–55]. Furthermore, changes in the cardiovascular and cerebrovascular systems along with induction of vascular inflammation and endothelial dysfunction may contribute to the onset of clinical cognitive decline and dementia. Aβ plaques are associated with upregulation of IL-1, IL6, and TNF-α, and levels correlate with disease severity [52]. Demyelination is also common in patients with heart disease [56], and reduction in axonal integrity of several brain circuits that are involved in cognition are found in these patients [57]. Dementia patients are more prone to structural and functional cardiac abnormality when compared with controls if they have suffered from heart disease previously [58].

## Phytochemicals in Herbs

Increasing evidence indicates that consumption of bioactive compounds or phytochemicals in herbs produces antioxidant, anti-inflammatory, and anti-carcinogenic effects (fig. 2). Unlike food products such as sugars, amino acids, and lipids, which are absorbed efficiently, the bioavailability of phytochemicals is largely poor, since a large proportion undergoes conjugation in the liver and is rapidly excreted. The low levels of phytochemicals that are absorbed are, however, able to exert biochemical effects by inhibiting activities of cyclooxygenases and lipoxygenases and stimulating activities of a number of protein kinases, protein phosphatases, and lipid kinases [59–61]. There are at least two major molecular mechanisms associated with the beneficial effects of phytochemicals—downregulation of the transcription factor, NF-κB, and upregulation of the transcription factor nuclear factor erythroid-2 (NrF2) [59]. Some phytochemicals stimulate the synthesis of adaptive enzymes and proteins that favor resistance to cellular stress (detoxifying and antioxidant enzymes). They increase the activities of antioxidant enzymes (superoxide dismutase, glutathione peroxidase, and catalase) and reduce the levels of 8-hydroxy-2′-deoxyguanine, resulting in decreased DNA damage [59, 62]. Phytochemicals also modulate angiogenesis and ionic homeostasis, and up-regulate the redox status of cells through signaling networks that control energy metabolism [59, 63]. Other phytochemicals interact with metal ions (iron and other transition metal ions) to form chemical complexes. The binding of phytochemicals with iron retards the Fenton reaction, thereby inhibiting free radical generation [64]. Based on their cardioprotective, nephroprotective, and neuroprotective properties of phytochemicals, it is proposed that long-term consumption of phytochemicals may retard or prevent oxidative stress and inflammation and lead to the delaying or slowing the onset of heart disease, stroke, AD, and CKD [59]. However, many present-day herbal remedies have yet to undergo careful scientific assessment, and some herbs may produce serious toxic effects and major drug interactions. Patients must therefore inform their healthcare professionals of everything they are taking, including medications, supplements, and ”natural products,” and healthcare professionals should be aware of the effects of herbs on health.

## Rationale for the Use of Multi-herb Formulas in TCM

Multi-herb formulas rather than single herbs are common in TCM medication. Each herb in a formula has a specific role—sovereign (君), minister (臣), assistant (佐), and courier (使). Sovereign and minister herbs treat the main symptoms and have a major role in the formula. Assistant herbs assist the sovereign and minister herbs to treat the accompanying symptoms, or reduce the side effects of the major herbs. Courier herbs help to lead the other components to the affected area. Interactions between the herbs, such as mutual reinforcement, antagonism, or detoxification, help to determine the formula’s therapeutic efficacy. The nature of the herbs, including the four properties (cold, hot, warm, and cool) and the five tastes (sour, sweet, bitter, acrid, and salty) as well as characteristics such as meridian-tropism, are taken into consideration by TCM physicians when formulating a prescription [65].

## Dementia and its Treatment as Recorded in Jingyue Quanshu (Collected Works of Zhang Jingyue)

Zhang Jing-Yue 张景岳 (c.1563–1640) had a great influence on the development of TCM, towards the end of the Ming Dynasty in China. He was born in Shaoxing County of Zhejiang Province, the birthplace of many of the country’s most renowned scholars and writers. Zhang traveled with his father to Beijing, where he studied medicine, and became an imperial physician working for Emperor Wanli. Because he made good use of processed Rehmannia glutinosa, he was also called “Zhang Shudi.” He was an outstanding medical scientist, the representative of the ancient Chinese medicine practice of “warm tonification,” and his academic thought has a great influence on future generations. A chapter on dementia (痴呆) in the Ming dynasty book 《Jingyue Quanshu》 (景岳全书 Collected Works of Zhang Jingyue; 1637 A.D.) describes how the collapse of original qi, together with the presence of impure qi in the meridians and heart orifices, can lead to the problem of dementia.

In this chapter, Zhang Jing-Yue was of the opinion that “dementia syndrome is characterized by lack of sputum, or stagnation, or failure, or anxiety, or suspicion, or panic, gradually leading to dementia. Speech and words are in reverse order and movement is sluggish. There is excessive sweating and depression. The symptoms may be extremely unusual and bizarre. The pulse is stringy or increased in frequency, large or small, and often changes. Persons have pathological qi in the heart and liver and biliary systems. The qi is unclear. If the body is strong and there is no reduction in food intake or other weaknesses, it is appropriate to treat using Fu Man Jian. It is the most stable and wonderful prescription. Nevertheless, some cases will recover and some will not, depending on the strength or weakness of stomach qi and original qi. Time is needed for recovery, which cannot be speeded up. Those who have this syndrome may be exacerbated by anxiety or depression leading to absent-mindedness and confusion. In this case, it is important to quickly support the good qi, mostly through the use of Qi Fu Yin or Da Bu Yan”. < 明代《景岳全书》张景岳认为:“痴呆证,凡平素无痰,而或以郁结,或以不遂,或以思虑,或以疑贰,或以惊恐,而渐致痴呆。言辞颠倒,举动不经,或多汗,或善愁,其证则千奇万怪,无所不至。脉必或弦或数,或大或小,变易不常。此其逆气在心或肝胆二经,气有不清而然。但察其形体强壮,饮食不减,别无虚脱等证。则悉宜服蛮煎治之。最稳最妙。然此证有可愈者,有不可愈者,亦在乎胃气元气之强弱,待时而复,非可急也。凡此诸证,若以大惊猝恐,一时偶伤心胆,而致失神昏乱者。此当以速扶正气为主,宜七福饮,或大补元煎主之。> [66]

## Description of Qi Fu Yin Herbs in Chinese Pharmacopeias

Qi Fu Yin (seven blessings decoction, 七福饮) is a mixture of seven herbs, *Panax ginseng*, *Rehmannia glutinosa*, *Angelica polymorpha*, *Atractylodes macrocephala*, *Glycyrrhiza uralensis*, *Ziziphus jujube*, and *Polygala tenuifolia* (Table 1)*.*

**Table:** 

No.	Order	Family	Genus	Species	Chinese name	Common name
1	Apiales	Araliaceae	*Panax*	*ginseng*	人参	Ginseng
2	Lamiales	Orobanchaceae	*Rehmannia*	*glutinosa*	熟地黄	Cooked Chinese Foxglove
3	Apiales	Apiaceae	*Angelica*	*polymorpha*	当归	Chinese Angelica root
4	Asterales	Asteraceae	*Atractylodes*	*macrocephala*	白朮	
5	Fabales	Fabaceae	*Glycyrrhiza*	*uralensis*	炙甘草	Roasted Licorice root
6	Rosales	Rhamnaceae	*Ziziphus*	*jujuba*	酸枣仁	Red date
7	Fabales	Polygalaceae	*Polygala*	*tenuifolia*	远志	Senega root

### *Panax ginseng*

According to one of three foundation books for TCM, Shennong Materia Medica, which is a compilation of oral traditions between 200 and 250 A.D., and attributed to a “divine farmer” Shennong, said to have lived around 2800 B.C.: ginseng has a sweet taste, and mildly cold in nature. It is mainly used for supplementing the five internal organs. It calms the spirit, stabilizes the soul, reduces fear, dispels impure qi, brightens the eyes, increases happiness, and is beneficial for thinking and wisdom. Long-term consumption leads to lightening of the body and extension of years. Another name is Human Title or Ghost Cover. It grows in valleys. < 神农本草经:人参,味甘微寒,主补五脏,安精神,定魂魄,止惊悸,除邪气,明目,开心益智。久服,轻身延年。一名人衔,一名鬼盖。生山谷。> [67]

According to *Bencao Gangmu* or *Compendium of Materia Medica*, a Chinese materia medica written by Shi-Zhen Li during the Ming dynasty in 1578 AD: ginseng supplements the five internal organs. It calms the spirit, stabilizes the soul, reduces fear, dispels impure qi, brightens the eyes, increases happiness, and is beneficial for thinking and wisdom. Long-term consumption leads to lightening of the body and extension of years. < 本草纲目:补五脏,安精神,定魂魄,止惊悸,除邪气,明目开心益智。久服轻身延年。> [68]. As can be seen above, it is very interesting that the descriptions of ginseng in Chinese Pharmacopeias have not changed in more than 1300 years, and that most of its beneficial effects are related to the brain. For a modern review on this subject, please see [69].

Usage and dosage: for oral consumption, as a decoction 3–10 g. If using a high dose of 10–30 g, should be prepared first as a single decoction and poured into the final prescription herbs. It can also be ground as a powder 1–2 g; or paste; or soaked in liquor/wine, or made into pills. < 用法用量:内服:汤剂,3-10克,大剂量10-30克,宜另煎兑入;或研末,1-2克;或敷膏;或泡酒;或入丸散。>

Contraindications for *Panax ginseng*: not to be consumed by persons with no deficiency, heat syndrome, or no weakness in qi. < 实证、热证而正气不虚者忌服。>

### *Rehmannia glutinosa*

According to Shen Nong’s Herbal Classic: Rehmannia has a sweet and cold taste and is non-toxic. It is mainly used for treatment of fractures and muscle injury, internal injury, dispelling blood stasis, filling of the bone marrow, and enhancing muscle growth. As a decoction, it helps to remove the accumulation of cold and heat, and remove paralysis. The raw herb is better. Long-term consumption leads to lightening of the body and anti-aging. < 神农本草经:地黄,甘、寒,无毒。主折跌绝筋,伤中,逐血痹,填骨髓,长肌肉,作汤除寒热积聚,除痹,生者尤良。久服轻身不老。> [67]

According to Compendium of Materia Medica: it fills the bone marrow, enhances muscle growth, increases essence and blood, supplements the five internal organs after internal injury, unblocks the blood circulation, is beneficial to the ears and eyes, and blackens the beard and hair. < 本草纲目:填骨髓,长肌肉,生精血,补五脏内伤不足,通血脉,利耳目,黑须发。> [68]

Usage and dosage: for oral consumption as a decoction 10–30 g. Or make into pills, pastes, or soak in wine/liquor < 用法用量:内服:煎汤,10-30克,或入丸散;或敷膏;或浸酒。>

Contraindications for Rehmannia glutinosa: persons with weakness in stomach, qi stagnation, abundant sputum, abdominal distension, and loose stools < 胃虚弱,气滞痰多,腹满便溏者忌服。>

### *Angelica polymorpha*

According to Shen Nong’s Materia Medica: Angelica has a bitter taste. It is warm in nature and non-toxic. It is mainly used for treatment of cough, reversed flow of qi, warm malaria, cold and hot contacts in the skin, women with uterine bleeding and infertility, all sore ulcers; and sores. Boil the herb to drink. <神农本草经:当归:苦、温,无毒。主咳逆上气,温瘧,寒热洗洗在皮肤中,妇人漏中绝子,诸恶疮疡,金疮。煮汁饮之 。> [67]

Usage and dosage: fried black, grind fine powder, each time use of 9 g, add a cup of water, a little wine, and decoction. 6~12 g. < 用法用量:炒黑,共研细末,每用9克,水一杯,酒少许,煎服。6~12克。>

Contraindications for *Angelica polymorpha*: excessive use causes tiredness, drowsiness, and other reactions. Stopping the medicine leads to disappearance of these symptoms. Allergic reaction: not suitable for persons with menorrhagia, bleeding tendency, yin deficiency, internal heat, and loose stools or diarrhea. Not to be consumed by persons with hot bleeding tendencies. To be consumed with caution by persons with wet fullness and bloating. < 用量过大有疲倦嗜睡等反应,停药消失。过敏反应月经过多,有出血倾向,阴虚内热,大便溏泄者均不宜服用。热盛出血患者禁服。湿盛中满慎服。>

### *Atractylodes macrocephala*

There is a saying that “ginseng in the north and Atractylodes in the south,” suggesting that Atractylodes is almost as highly regarded as ginseng in TCM. According to Shen Nong’s Herbal Classic: Atractylodes macrocephala has a bitter taste and warm nature. It is mainly used for treatment of rheumatism, joint ache caused by wind and dampness, muscle paralysis, muscle spasm, and jaundice. It stops perspiration, dispels heat, helps digestion, and is used in decoctions. Long-term consumption leads to lightening of the body, extension of years, and satiety. Another name is mountain thistle, it grows in valleys. < 神农本草经:白术:味苦温。主风寒湿痹死肌,痉疸,止汗,除热,消食,作煎饵。久服轻身延年,不饥。一名山蓟,生山谷。> [67].

Usage and dosage: 6~12 g.< 用法用量:6~12克。>

Contraindications for Atractylodes macrocephala: not to be consumed by persons with Yin deficiency, qi stagnation, and nausea < 阴虚燥渴,气滞胀闷者忌服。>

### *Glycyrrhiza uralensis* (Licorice Root)

According to Shen Nong’s Herbal Classic: *Glycyrrhiza uralensis* has a sweet taste and a neutral nature. It is mainly used to dispel cold and hot, impure qi in the five viscera (heart, spleen, liver, lungs, and kidneys) and six hollow organs (gallbladder, stomach, large intestine, small intestine, bladder, and sanjiao 三焦), toughen tendons and bones, increase muscle mass, multiply strength, facilitate recovery after traumatic injury, and detoxification. Long-term consumption leads to lightening of the body and extension of years. < 神农本草经:炙甘草:味甘,平。主五脏六腑寒热邪气,坚筋骨,长肌肉,倍力,金疮尰,解毒。久服轻身延年。> [67]

Usage and dosage: 1.5~9 g. < 用法用量:1.5~9克。>

Contraindications for *Glycyrrhiza uralensis*: not appropriate to combine with Jing Da Ji (Radix Euphorbiae), Yuan Hua (Lilac Daphne Flower Bud), and Gan Sui (Radix KanSui) together. < 不宜与京大戟,芫花,甘遂同用。>

### *Ziziphus jujube* (Red Dates)

According to Shen Nong’s Materia Medica, it is mainly used to treat heart and abdominal cold and heat, and aggregation of impure qi; aching, pain; and dampness arthralgia. Long-term consumption leads to calming of the five viscera (heart, spleen, liver, lungs, and kidneys), lightening of the body, and extension of years. < 神农本草经:主心腹寒热,邪结气聚,四肢酸痛湿痹,久服安五脏,轻身延年。> [67].

According to Compendium of Materia Medica, the seed has a sweet taste and a neutral nature. The processed herb is used for treatment of biliary system weakness, insomnia, polydipsia, and sweating. Its raw form is used for treatment of heaty biliary system and accompanying drowsiness. It is also the drug for treatment of the diseased accupoints: liver meridian of foot—Jueyin, and gallbladder meridian of foot—Shaoyang. < 本草纲目:“其仁甘而润,故熟用疗胆虚不得眠,烦渴虚汗之证;生用疗胆热好眠,皆足厥阴、少阳药也。” > [68]

Usage and dosage: put into the decoction, 9~15 g. < 用法用量:入汤剂,9~15克。>.

Contraindications for Ziziphus jujube: to be consumed with caution by persons with impure smoldering fire and those with chronic diarrhea. Not to be used with Fang Ji (Radix Stephaniae tetrandrae). < 凡有实邪郁火及患有滑泄症者慎服。恶防己。>.

### *Polygala tenuifolia*

According to Shen Nong’s Herbal Classic: Polygala has a bitter taste and a warm nature. It is mainly used to treat cough with dyspnea, internal injury, make up for deficiency, dispel impure qi, and relieve obstruction in the nine orifices (ears, eyes, mouth, nose, urethra, and anus). It is beneficial for wisdom, and makes one clever and not forgetful. It strengthens ambition and resolve, and multiples strength. Long-term consumption leads to lightening of the body and anti-aging. The name of the name of the leaf is Small Grass. Another name is Spine Wan (Lu De Ming Er Ya Yin Sheng cited amaranth). Another name is Spine Around (Royal View Around). Another name is Fine Grass. It grows in valleys. < 神农本草经:远志:味苦温。主咳逆,伤中,补不足,除邪气,利九窍,益智慧,使人聪明,不忘,强志倍力。久服,轻身不老。叶名小草,一名棘菀(陆德明尔雅音义引作苋),一名棘绕(御览作要绕),一名细草。生川谷。> [67]

According to compendium of Materia Medica: it has a bitter taste and warm nature and non-toxic. It is mainly used to treat forgetfulness. Take Polygala at the end and boil with water to consume < 本草纲目:苦、温、无毒。「主治」善忘症。取远志为末,冲服。> [68]

Usage and dosage: boil and consume: 3~9 g. < 用法用量:煎服,3~9克。>

Contraindications for Polygala tenuifolia: persons with gastritis and gastric ulcer should use with caution. < 有胃炎及胃溃疡者慎用。>

## Indications, Contraindications, and Possible Adverse Effects of Qi Fu Yin

### Indications for Qi Fu yin

Qi Fu Yin is used for treatment of heart and qi deficiency, treatment of neurasthenia, and treatment of age-related dementia. It has effects on benefitting qi, supplementing the blood, nourishing the heart, and calming the spirit. < 治疗心气虚,治疗神经衰弱,治疗老年性痴呆。有益气补血,养心宁神的功效。> [66, 67]. *Panax ginseng* (with the heart, sovereign herb), tonifying qi and lifting yang, is beneficial for happiness, thinking and wisdom, strengthens the spleen and nourishes the stomach < 人参(随宜心,君),补气补阳,开心益智,健脾养胃 > [66, 67] . *Atractylodes macrocephala* (along with the lungs, minister herb), strengthens the spleen, reduces dampness, benefits qi, and helps in the circulation < 白术(随宜肺,臣),健脾燥湿,加强益气助运之力 > [66, 67]. *Rehmannia glutinosa* (with the *kidney*, minister) (In YJW’s opinion, could be a second sovereign herb, present in some TCM formulas), *Angelica polymorpha* (with the liver, minister), *Ziziphus jujube* (assistant), and *Polygala tenuifolia* (assistant) boost the blood, calms the mind, and calms the spirit. < 熟地(随宜肾,臣)、当归(随宜肝,臣)、枣仁(佐)、远志(佐),补血宁心安神。> [66, 67] *Glycyrrhiza uralensis* (with the spleen, courier) is beneficial to qi, nourishes the heart, and harmonizes the different medicines, thus leading to filling of the qi and blood and calming of the mind and spirit, resulting in recovery from disease. < 炙甘草(随宜脾,使),益气和中养心,调和诸药,从而使气血充、心神安则病愈。> [66, 67]

### Contraindications for Qi Fu Yin

Persons who have heat syndrome and no weakness in qi should not consume. Women should stop using it during menstruation < 实证、热证而正气不虚者忌服。妇女经期停用。> [66, 67].

### Adverse Effects of Qi Fu Yin

None recorded to our knowledge.

## Preparation and Use of Qi Fu Yin

The following herbs are boiled in 400 cc of water and left to simmer until the volume reduces to 280 cc. This can be divided into 2 servings per day, to be taken on an empty stomach, when warm. <上药用水400毫升,煎取280毫升,空腹时温服。> [66]. Review in 1-3 months.

**Table:** 

No.	Genus	Species	Chinese name	Weight in original prescription	Weight in modern prescription
1	*Panax*	*ginseng*	人參	6 g	6 g
2	*Rehmannia*	*glutinosa*	熟地黄	9 g	9 g
3	*Angelica*	*polymorpha*	當歸	6-9 g	9 g
4	*Atractylodes*	*macrocephala*	白朮(炒)	4.5 g	5 g
5	*Glycyrrhiza*	*uralensis*	炙甘草	3 g	3 g
6	*Ziziphus*	*jujuba*	酸棗仁	6 g	6 g
7	*Polygala*	*tenuifolia*	遠志 (制用)	0.9–1.5 g	5 g

## Conclusion

Alzheimer’s disease and vascular dementia are two common causes of dementia, and it is increasingly recognized that many older adults with dementia have both AD and vascular pathologies. The underlying molecular mechanisms are incompletely understood, but may involve atherosclerosis, vascular dysfunction, hypertension, type 2 diabetes, history of cardiac disease, and total homocysteine, and possibly, lack of erythropoietin formation by the kidney. During the Ming Dynasty, Zhang Jing-Yue used Qi Fu Yin (seven blessings decoction), comprising *Panax ginseng*, *Rehmannia glutinosa*, *Angelica polymorpha*, *Atractylodes macrocephala*, *Glycyrrhiza uralensis*, *Ziziphus jujube*, *and Polygala tenuifolia* to boost qi and blood circulation, strengthen the heart, and calm the spirit—skillfully linking heart, spleen, kidney, qi, blood and brain as a whole to treat age-related dementia.
